# Functional and Disability Assessment Among Hispanics With Zone 2 Flexor Tendon Injuries: Comparative Study Between Flexor Digitorum Superficialis Repair and Flexor Digitorum Superficialis Excision

**DOI:** 10.5435/JAAOSGlobal-D-20-00081

**Published:** 2020-09-01

**Authors:** Eduardo J. Natal-Albelo, Gerardo Olivella, Giovanni U. Paraliticci-Márquez, Lenny Rivera, Gabriel Echegaray, Norman Ramírez, Christian A. Foy-Parrilla

**Affiliations:** From the Orthopedic Surgery Department, University of Puerto Rico (UPR) Medical Sciences Campus, San Juan, Puerto Rico (Dr. Natal-Albelo, Dr. Olivella, Dr. Paraliticci-Márquez, Dr. Rivera, Dr. Echegaray, and Dr. Foy-Parrilla), and the Pediatric Orthopedic Surgery Department, Mayagüez Medical Center, Mayagüez, Puerto Rico (Dr. Ramírez).

## Abstract

Flexor tendon lacerations in zone II have been reported to be the most complicated of all tendon injuries. Currently, there is no consensus on treatment in surgical management for patients with flexor tendon laceration of flexor digitorum profundus and flexor digitorum superficialis (FDS). The aim of this study was to evaluate whether the repair of FDS tendons provided superior functional outcomes compared with FDS excision in Hispanic patients.

**Methods:**

Total active motion, original Strickland criteria, and the disability of arm shoulder and hand questionnaire were provided postoperatively at 3 and 6 months to all consecutive Hispanic patients who underwent zone II flexor tendon repair. The cohort was divided into two groups, those who underwent FDS repair and those underwent FDS excision.

**Results:**

Functional and disability outcome analysis showed a notable improvement with FDS repair using total active motion, Strickland criteria, and disability of arm shoulder and hand score at the 3 months postoperative interval. No statistical differences were identified regarding functional and disability outcomes at the 6-month evaluation between both groups.

**Conclusions:**

Among Hispanics, the FDS-repaired group had similar functional and disability outcomes at their 6 months postoperative evaluation compared with the FDS-excised group. Increased awareness for tendon rerupture during the initial 3 months of index surgery is recommended for FDS-excised patients.

A zone II flexor tendon injury is defined as a traumatic condition that advances from the proximal aspect of the annular (A1) pulley to the insertion of the flexor digitorum superficialis (FDS) tendon at the middle phalanx.^1,2^ The uniqueness of this injury lies in the restoration of tendon gliding within a tight fibro-osseous sheath that covers both flexor digitorum profundus (FDP) and FDS tendons while minimizing the formation of adhesions in surrounding tissues.^2,3^ Currently, there is a consensus that zone II flexor tendon lacerations must undergo primary surgical repair, yet there is still controversy on the type of surgical repair approach.^3-5^ Some authors advocate that the sole repair of the FDP tendon with excision of the FDS tendon can help minimize tendon gliding by reducing the bulk of the repaired tendon.^6,7^ However, other authors prefer to repair both FDP and FDS tendon slips depending on the smoothness of intraoperative tendon gliding.^8^

In agreement with any other type of trauma, proper healing of zone II injuries is influenced by multiple factors: ranging from access to healthcare, risk occupations, mechanism of injury, functional disability, and ethnicity, among other problems.^9,10^ The Hispanic population have been categorized as a minority group that is almost two times more likely to develop upper extremity injuries involving the hand compared with a non-Hispanic population.^10^ Hispanics were also found to be less adherent to postoperative protocols than non-Hispanics after these types of injuries.^11^

The surgical literature evaluating primary surgical repair of FDP alone with FDS excision versus the repair of both FDP and FDS in patients with zone II hand injuries is limited.^4,5,12^ To our knowledge, studies that show the functional and disability outcomes of Hispanic patients with zone II flexor tendon injuries have never been reported. Therefore, the aim of this study was to evaluate if the repair of FDP and FDS tendons could improve the functional outcomes when compared with FDP repairs and excisions of FDS tendons in Hispanic patients with zone II flexor tendon injuries. We hypothesized that at the 6 months postoperative follow-up, Hispanic patients with zone II flexor tendon injuries who undergo FDP and FDS repair will present better functional outcomes and lower disability scores.

## Methods

A prospective cohort observational study of all Hispanic patients who suffered zone II flexor tendon lacerations was performed in a state-designated level one trauma medical center from March 2016 to March 2018. The cohort was categorized into two groups: those who received the repair of both FDP and FDS tendons (FDS-repair group) and those who received FDP repair with excision of FDS (FDS-excision group). A surgical alternate allocation was based on calendar days, with FDS-repair group performed on even days and FDS-excision group on uneven days. The protocol performed in this study was reviewed and approved by the Institutional Review Board of the University of Puerto Rico Medical Sciences Campus.

All patients older than 18 years of age who presented to the emergency department with isolated laceration of flexor tendons of the hand at zone II were invited to participate in the study. Informed consent was signed by every patient enrolled in the study who was willing to return for at least a 6-month postoperative follow-up evaluation. Patients who were (1) younger than 18 years old, (2) had phalangeal fractures, (3) had complex and extensive soft-tissue damage, (4) had partial or total rupture of A2 pulley at the time of surgery, (5) had injury of contralateral hand, (6) had neurologic injury, or (7) had inability to follow postoperative protocol were excluded from the study.

A single fellowship-trained orthopaedic hand surgeon (PI) performed all surgeries at a mean time of 14 days (range 8 to 19 days) after the initial hand injury accident. All surgeries were performed with a regional block anesthesia. Brunner incisions and midaxial approach were used for tendon exposure.^13^ The digital injured sheath was opened proximal to the A2 pulley. The FDP and FDS tendon lacerations were repaired using four stranded locked cruciate techniques with 3-0 Ti-Cron polyester braided for core sutures, and augmentation was performed with continuous interlocking epitendinous repair using Prolene 6-0. The digits where the FDS tendon was excised, a resection proximal to the A2 pulley, was performed.

Postoperatively, patients were immobilized with dorsal blocking splint with the wrist in 10° of flexion, metacarpal phalangeal (MCP) joint in 70° of flexion, and interphalangeal joints in full extension for 1 week.^14^ Patients were evaluated for this protocol at first week, third month, and sixth month postoperatively in our outpatient orthopaedic hand clinics. During the first postoperative visit, the surgical compressive dressings were removed, wounds were evaluated, and covered with a light compressive dressing to the hand & forearm. After the first postoperative visit, all patients began a standard rehabilitation protocol given by a single certified hand occupational therapist. The modified Duran place and hold flexor rehabilitation protocol was chosen as the preferred protocol, which was completed by all patients in an 8-weeks period.^14,15^

Per protocol, at the third and sixth months, the progress was evaluated through three systems: (1) the total active motion (TAM) score, (2) the original Strickland criteria, and (3) the quick disabilities of the arm, shoulder and hand (DASH) questionnaire.^15,16,17,18,19^ All of the outcomes were recorded and charted blindly by the occupational therapist. Goniometric measurements of MCP, proximal interphalangeal (PIP), and distal interphalangeal (DIP) joint flexion were documented after each patient performed a fist and extended the digit of interest. First, the TAM score was calculated with the sum of the degrees of active MCP, PIP, and DIP joint flexion minus the degrees from full extension of the healthy contralateral digit.^16^ A score of 260° was defined as a normal tendon flexion TAM score.^17^ Second, the original Strickland criteria outcomes were based on PIP and DIP joint degree movement from poor to excellent results.^15^ Poor results were given to a PIP and DIP range of motion (ROM) of less than 90° (less than 50% of normal value).^15^ Fair results had a functional ROM from 90° to 124° (50% to 69% of normal value).^15^ Good results had a ROM between 125° and 149° (70% to 84% of normal value).^15^ Excellent results were assigned to a ROM greater than 150° (85% to 100% of normal value).^15^ Finally, the DASH self-questionnaire (Spanish validated version) was given to all patients with the purpose to measure self-rated upper extremity disability or symptoms.^18,19^ The DASH questionnaire consists of a 30-item disability and symptom scale that centers on three aspects: the degree of difficulty of physical activity because of arm, shoulder, or hand problems (21 items), severity of their symptoms (pain, activity-related pain, tingling, weakness, and stiffness; 5 items), and their impact on their daily life (4 items).^18^ This questionnaire is scored from 0 (no disability) to 100 (severe disability).^18^

Postoperative complications were documented for each case, such as tendon ruptures. The frequency of revision procedures, along with the indication for revision and surgical revision procedures performed were also obtained and charted.

Demographics such as age, sex, mechanism of injury, and digits involved, along with postoperative complications, TAM, and DASH score, were evaluated between both groups.^4^ The differences among both groups studied about age, TAM, and DASH score were analyzed with a Student *t*-test. A Fischer exact test was used to compare and analyze the original Strickland classification scores and postoperative complications between both groups. An alpha of 0.05 with a confidence interval of 95% was used, establishing a *P* ≤ 0.05 for statistical significance. Microsoft Excel and GraphPad Prism-8 were used for comparison and analysis of the variables studied.

## Results

A total of 25 digits of 18 consecutive Hispanic patients who underwent flexor tendon repair due to traumatic zone II hand laceration met the inclusion criteria for the study. There were 16 female digits (64.0%) and 9 male digits (36.0%); with an average group age of 38.6 ± 13.1 years. Eleven digits (44.0%) underwent repair of both FDP and FDS tendons (FDS-repair group), and a total of 14 digits (56.0%) underwent FDS-excision with repair of FDP tendon (FDS-excision group). At the time of injury, unemployment was the most common occupation (13 digits involved), followed by office worker (8 digits involved) and manual worker (4 digits involved). Active smoking was reported on four digits (16.0%) in the whole group. The most common mechanism of injury were knife lacerations in 17 digits (68.0%), followed by saw injuries in 5 digits (20.0%), and glass-related lacerations in 3 digits (12.0%). Small digits were the most predominant flexor injured with 40% of the sample data. Of all patients, 16 digits (64.0%) were covered by government insurance and 9 digits (36.0%) were covered by private insurance. Comparison of the demographic data between both groups did not show any significant difference. Data reported on Tables 1–3, illustrates the analysis of the demographic information of zone II injuries stratified by digits, hand/patients, and mechanism of injury.

**Table 1:** Demographics of Digits With Zone II Injury Who Underwent Flexor Tendon Injuries

Variables Stratified by Digits	Total (N = 25)	FDS-Repair (N = 11)	FDS-Excision (N = 14)	*P* Value
Digits distribution by sex (no.)				
Male	9 (36.0)	3 (27.3)	6 (42.9)	0.68
Female	16 (64.0)	8 (72.7)	8 (57.1)	
Age (yr)	38.6 ± 13.1	44.5 ± 14.3	34.0 ± 18.3	0.13
Insurance plan (no.)				
Government	16 (64.0)	6 (54.5)	10 (71.4)	0.43
Private	9 (36.0)	5 (45.5)	4 (28.6)	
Comorbidities (no.)				
Hyperlipidemia	2 (8.0)	1 (9.1)	1 (7.1)	1.00
Hypertension	4 (16.0)	3 (27.3)	1 (7.1)	0.29
Diabetes mellitus type-II	1 (4.0)	0 (0.0)	1 (7.1)	1.00
None	18 (72.0)	7 (63.6)	11 (78.6)	0.66
Smoker (no.)				
Yes	4 (16.0)	3 (27.3)	1 (7.1)	0.29
No	21 (84.0)	8 (72.7)	13 (92.9)	
Occupation (no.)				
Office worker	8 (32.0)	5 (45.4)	3 (21.4)	0.39
Manual worker	4 (16.0)	2 (18.2)	2 (14.3)	1.00
Unemployed	13 (52.0)	4 (36.4)	9 (64.3)	0.24
Mechanism of injury (no.)				
Knife/blade lacerations	17 (68.0)	8 (72.7)	9 (64.3)	1.00
Saw injury	5 (20.0)	1 (9.1)	4 (28.6)	0.34
Glass-related lacerations	3 (12.0)	2 (18.2)	1 (7.1)	0.57
Digits involved (no.)				
Index digits	4 (16.0)	2 (18.2)	2 (14.3)	1.00
Middle digits	4 (16.0)	2 (18.2)	2 (14.3)	1.00
Ring digits	7 (28.0)	3 (27.3)	4 (28.6)	1.00
Small digits	10 (40.0)	4 (36.4)	6 (42.9)	1.00

FDS = flexor digitorum superficialis

**Table 2:** Demographics of Hands With Zone II Injury Who Underwent Flexor Tendon Injuries

Variables Stratified by Hand	Total (N = 18 Hands)	FDS-Repair (N = 9 Hands)	FDS-Excision (N = 9 Hands)	*P* Value
Hand distribution by sex (No.)				
Male	7 (38.9)	3 (33.3)	4 (44.4)	1.00
Female	11 (61.1)	6 (66.7)	5 (55.6)	
Age (yr)	38.6 ± 13.1	44.5 ± 14.3	34.0 ± 18.3	0.13
Insurance plan (no.)				
Government	11 (61.1)	4 (44.4)	7 (77.8)	0.34
Private	7 (38.9)	5 (55.6)	2 (22.2)	
Comorbidities (no.)				
Hyperlipidemia	2 (11.1)	1 (11.1)	1 (11.1)	1.00
Hypertension	3 (16.7)	2 (22.2)	1 (11.1)	
Diabetes mellitus type-II	1 (5.6)	0 (0.0)	1 (11.1)	
None	12 (66.7)	6 (66.7)	6 (66.7)	
Smoker (no.)				
Yes	4 (22.2)	3 (33.3)	1 (11.1)	0.58
No	14 (77.8)	6 (66.7)	8 (88.9)	
Occupation (no.)				
Office worker	6 (33.3)	4 (44.4)	2 (22.2)	0.62
Manual worker	3 (16.7)	1 (11.1)	2 (22.2)	1.00
Unemployed	9 (50.0)	4 (44.4)	5 (55.6)	1.00
Mechanism of injury (no.)				
Knife/blade lacerations	11 (61.1)	6 (66.7)	5 (55.6)	1.00
Glass-related lacerations	3 (16.7)	2 (22.2)	1 (11.1)	1.00
Saw injury	4 (22.2)	1 (11.1)	3 (33.3)	0.58
Injured site (no.)				
Right	15 (83.3)	8 (88.9)	7 (77.8)	1.00
Left	3 (16.7)	1 (11.1)	2 (22.2)	
Digits involved per hand (no.)				
One digit	13 (72.2)	7 (77.8)	6 (66.7)	1.00
Two digits	3 (16.7)	2 (22.2)	1 (11.1)	1.00
Three digits	2 (11.1)	0 (0.0)	2 (22.2)	0.47

FDS = flexor digitorum superficialis

**Table 3:** Demographic of Digits With Zone II Injury Who Underwent Flexor Tendon Injuries Stratified by Mechanism of Injury

Variables	Saw Injury (N = 5)	Sharp Injury^a^ (N = 20)	*P* Value
Finger distribution by Sex (no.)			
Male	3 (60.0)	6 (30.0)	0.31
Female	2 (40.0)	14 (70.0)	
Age	34.0 ± 13.8	39.8 ± 13.0	0.39
Comorbidities (no.)			
Hyperlipidemia	1 (20.0)	1 (5.0)	0.37
Hypertension	0 (0.0)	4 (20.0)	0.55
Diabetes mellitus type-II	0 (0.0)	1 (5.0)	1.00
None	4 (80.0)	14 (70.0)	1.00
Smoker (no.)			
Yes	1 (20.0)	3 (15.0)	1.00
No	4 (80.0)	17 (85.0)	
Insurance plan (no.)			
Government	4 (80.0)	12 (60.0)	0.62
Private	1 (20.0)	8 (40.0)	
Occupation (no.)			
Office worker	1 (20.0)	7 (35.0)	1.00
Manual worker	1 (20.0)	3 (15.0)	1.00
Unemployed	3 (60.0)	10 (50.0)	1.00
Surgical intervention			
FDS repair	1 (20.0)	10 (50.0)	0.34
FDS excision	4 (80.0)	10 (50.0)	
Fingers involved (Qty)			
Small	4 (80.0)	6 (30.0)	0.12
Ring	1 (20.0)	6 (30.0)	1.00
Middle	0 (0.0)	4 (20.0)	0.55
Index	0 (0.0)	4 (20.0)	0.55

FDS = flexor digitorum superficialis

a Includes both knife/blade and glass-related lacerations.

The average ROM (TAM% score) for patients who underwent FDS-repair was 57.9% ± 12.5% at 3-months and improved to 76.2% ± 14.0% at 6-months evaluation. In the FDS-excision group, the average TAM score was 44.0% ± 13.5% at 3-months and improved to 73.4% ± 9.8% at 6-months evaluation. Three-months post-surgical evaluation revealed that patients who were part of the FDS-repair group reported superior ROM (TAM% score) compared with the FDS-excision group (*P* = 0.025). Interestingly, the comparison at 6-months post-surgery showed that the FDS-repair group reported better clinical ROM (TAM score) outcomes versus the FDS-excision group; although it was not found statistically significant (*P* = 0.62). Upon the evaluation of ROM by joints, the MCP (*P* = 0.019), PIP (*P* = 0.029) and DIP (*P* < 0.001) had better outcomes at 3-months visit in the FDS-repair group compared with the FDS-excision group. The evaluation at 6-months visit showed an overall group ROM improvement of all the joints except for the MCP at the FDS-repair group. No significant difference was noted between both groups at the 6-months follow-up. The functional results based on original Strickland's criteria showed that the overall group improved their scores at 6-months from their 3-months evaluation; where 11 digits had excellent results (*P* = 0.008) and 14 digits had good results (*P* = 0.154). None of the tendons evaluated had fair or poor results at their final 6-months follow-up. Similar results were found between both groups at the 3-months and 6-months follow-ups. The DASH score of the 3-months postoperative follow-up evaluation showed lower disability scores in the FDS-repair group with an average of 10.1 ± 7.0 compared with 23.3 ± 9.6 in the FDS-excision group (*P* < 0.001). At the 6-months follow-up, both groups had lowered their DASH scores from the previous 3-months visit with an average DASH score of 6.4 ± 3.2 in the FDS-repair group and 10.4 ± 6.2 in the FDS-excision group. Comparison of both groups at the 6-months follow-up did not show a significant difference on DASH score (*P* = 0.07).

The analysis of the functional outcomes of zone II flexor injuries in all digits, each digit involved, hand/patients, and mechanism of injury are illustrated in Tables 4–7, respectively. Two digits who underwent FDS excision required to be reoperated during their first 3-months follow-up period due to FDP tendon re-rupture. Both digits underwent FDP tendon revision and completed the rehabilitation protocol. None of the patients in the FDS-repair group had postoperative complications.

**Table 4:** Functional Outcomes of Digits With Zone II Injury Who Underwent FDS Repair or Excision

Variables Stratified by Digits	Total (N = 25)	FDS Repair (N = 11)	FDS Excision (N = 14)	*P* Value
Follow-up at 3 mo				
MCP (deg.)	86.6 ± 4.5	83.8 ± 7.4	89.4 ± 1.6	0.02
PIP (deg.)	59.7 ± 13.5	67.0 ± 10.4	52.3 ± 16.5	0.03
DIP (deg.)	24.1 ± 15.9	33.4 ± 21.5	14.8 ± 10.2	<0.01
TAM (%)	51.0 ± 13.0	57.9 ± 12.5	44.0 ± 13.5	0.03
Dash score (no.)	16.7 ± 8.3	10.1 ± 7.0	23.3 ± 9.6	<0.01
Strickland criteria				
Excellent (85-100)	2 (8.0)	2 (18.2)	0 (0.0)	0.18
Good (70-84)	8 (32.0)	3 (27.3)	5 (41.7)	1.00
Fair (50-69)	13 (52.0)	6 (54.5)	7 (50.0)	1.00
Poor (0-49)	2 (8.0)	0 (0.0)	2 (8.3)	0.49
Follow-up at 6 mo				
MCP (deg.)	88.6 ± 2.7	89.2 ± 2.9	88.0 ± 2.4	0.27
PIP (deg.)	75.2 ± 21.9	80.0 ± 24.4	70.4 ± 19.4	0.35
DIP (deg.)	37.4 ± 15.1	36.3 ± 18.1	38.5 ± 12.1	0.61
TAM (%)	74.8 ± 11.9	76.2 ± 14.0	73.4 ± 9.8	0.62
Dash score (no.)	8.4 ± 3.2	6.4 ± 3.2	10.4 ± 6.2	0.07
Strickland criteria				
Excellent (85-100)	11 (44.0)	5 (45.5)	6 (41.7)	1.00
Good (70-84)	14 (56.0)	6 (54.5)	8 (58.3)	1.00
Fair (50-69)	0 (0.0)	0 (0.0)	0 (0.0)	1.00
Poor (0-49)	0 (0.0)	0 (0.0)	0 (0.0)	1.00

DIP = distal interphalangeal, FDS = flexor digitorum superficialis, MCP = metacarpal phalangeal, PIP = proximal interphalangeal, TAM = total active motion

**Table 5:** Functional Outcomes of Zone II Injuries Who Underwent FDS Repair or Excision by Each Digit

Variables	Small Finger			Ring Finger			Middle Finger			Index Finger		
	FDS Repair (N = 4)	FDS Excision (N = 6)	*P* Value^a^	FDS Repair (N = 3)	FDS Excision (N = 4)	*P* Value	FDS Repair (N = 2)	FDS Excision (N = 2)	*P* Value^a^	FDS Repair (N = 2)	FDS Excision (N = 2)	*P* Value
Follow-up at 3 mo												
MCP (deg.)	84.0 ± 4.1	88.6 ± 2.8	0.06	84.3 ± 6.4	89.0 ± 1.4	0.20	86.4 ± 8.6	89.5 ± 0.7	0.66	80.4 ± 10.5	89.0 ± 1.4	0.37
PIP (deg.)	78.3 ± 9.1	50.5 ± 10.8	<0.01	65.5 ± 10.4	46.4 ± 21.0	0.21	60.2 ± 11.9	55.3 ± 1.4	0.62	63.1 ± 10.3	56.8 ± 31.8	0.81
DIP (deg.)	39.1 ± 16.5	14.4 ± 10.4	0.02	31.7 ± 26.5	13.6 ± 9.6	0.25	30.3 ± 21.7	16.0 ± 10.1	0.49	31.3 ± 19.4	15.3 ± 11.2	0.42
TAM (%)	78.6 ± 10.3	52.3 ± 7.0	<0.01	57.7 ± 15.2	39.6 ± 7.2	0.09	47.8 ± 13.2	39.0 ± 9.0	0.52	47.4 ± 11.4	45.0 ± 30.9	0.93
Dash score (no.)	9.1 ± 4.9	12.9 ± 9.5	0.49	9.8 ± 5.8	23.0 ± 10.6	0.11	11.1 ± 6.4	27.7 ± 8.2	0.15	10.5 ± 8.9	29.7 ± 9.8	0.18
Strickland criteria												
Excellent (85-100)	0 (0.0)	0 (0.0)	1.00	0 (0.0)	0 (0.0)	1.00	1 (50.0)	0 (0.0)	1.00	1 (50.0)	0 (0.0)	1.00
Good (70-84)	2 (50.0)	2 (33.3)		1 (33.3)	2 (50.0)		0 (0.0)	0 (0.0)		0 (0.0)	1 (50.0)	
Fair (50-69)	2 (50.0)	3 (50.0)		2 (66.7)	2 (50.0)		1 (50.0)	2 (100.0)		1 (50.0)	0 (0.0)	
Poor (0-49)	0 (0.0)	1 (16.7)		0 (0.0)	0 (0.0)		0 (0.0)	0 (0.0)		0 (0.0)	1 (50.0)	
Follow-up at 6 mo												
MCP (deg.)	89.2 ± 1.2	87.0 ± 2.8	0.18	90.0 ± 2.5	89.0 ± 1.4	0.53	88.5 ± 4.5	87.5 ± 3.8	0.83	88.7 ± 3.2	89.0 ± 1.4	0.91
PIP (deg.)	81.0 ± 22.6	78.4 ± 19.5	0.85	82.6 ± 23.8	55.0 ± 25.7	0.21	72.5 ± 26.4	70.0 ± 14.1	0.92	84.2 ± 24.8	78 ± 17.0	0.80
DIP (deg.)	42.3 ± 17.1	44.2 ± 11.6	0.84	34.0 ± 19.5	33.6 ± 12.7	0.98	34.1 ± 20.4	36.7 ± 16.1	0.90	34.6 ± 15.3	38.0 ± 8.2	0.81
TAM (%)	88.6 ± 12.1	80.5 ± 8.6	0.25	74.5 ± 17.8	65.9 ± 8.1	0.42	69.0 ± 15.2	71.4 ± 9.3	0.87	72.6 ± 11.4	75.9 ± 13.1	0.81
Dash score (no.)	5.5 ± 2.1	8.7 ± 4.2	0.20	6.3 ± 3.7	10.2 ± 5.4	0.34	6.2 ± 4.1	12.1 ± 6.4	0.39	7.7 ± 3.2	10.6 ± 8.9	0.71
Strickland criteria												
Excellent (85-100)	2 (50.0)	2 (33.3)	1.00	1 (33.3)	1 (25.0)	1.00	1 (50.0)	1 (50.0)	1.00	1 (50.0)	2 (100.0)	1.00
Good (70-84)	2 (50.0)	4 (66.7)		2 (66.7)	3 (75.0)		1 (50.0)	1 (50.0)		1 (50.0)	0 (0.0)	
Fair (50-69)	0 (0.0)	0 (0.0)		0 (0.0)	0 (0.0)		0 (0.0)	0 (0.0)		0 (0.0)	0 (0.0)	
Poor (0-49)	0 (0.0)	0 (0.0)		0 (0.0)	0 (0.0)		0 (0.0)	0 (0.0)		0 (0.0)	0 (0.0)	

DIP = distal interphalangeal, FDS = flexor digitorum superficialis, MCP = metacarpal phalangeal, PIP = proximal interphalangeal, TAM = total active motion

**Table 6:** Functional Outcomes of Digits With Zone II Injury Who Underwent Repair Stratified by Number of Digits per Hand Involved

Variables by Hand	Total	One Digit	Multiple Digits	*P* Value
	(N = 18 Hands)	(N = 13 Hands)	(N = 5 Hands)	
Follow-up at 3 mo				
MCP (deg.)	86.6 ± 4.5	86.5 ± 5.4	86.7 ± 3.5	0.08
PIP (deg.)	59.7 ± 13.5	60.6 ± 10.4	58.8 ± 16.6	0.78
DIP (deg.)	24.1 ± 15.9	30.8 ± 17.4	17.2 ± 14.4	0.14
TAM (%)	51.0 ± 13.0	53.3 ± 13.3	48.6 ± 12.7	0.51
Dash score (no.)	16.7 ± 8.3	17.5 ± 7.5	15.9 ± 9.1	0.71
Strickland criteria				
Excellent (85-100)	1 (5.6)	0 (0.0)	1 (20.0)	0.28
Good (70-84)	5 (27.8)	3 (23.1)	2 (40.0)	0.58
Fair (50-69)	10 (55.6)	8 (61.5)	2 (40.0)	0.61
Poor (0-49)	2 (11.1)	2 (15.4)	0 (0.0)	1.00
Follow-up at 6 mo				
MCP (deg.)	88.6 ± 2.7	89.1 ± 4.0	88.0 ± 1.3	0.56
PIP (deg.)	75.2 ± 21.9	77.4 ± 20.3	73.0 ± 23.4	0.70
DIP (deg.)	37.4 ± 15.1	38.0 ± 19.5	36.7 ± 10.7	0.89
TAM (%)	74.8 ± 11.9	76.0 ± 13.4	73.6 ± 10.5	0.73
Dash score (no.)	8.4 ± 3.2	9.5 ± 2.5	7.3 ± 3.8	0.17
Strickland criteria				
Excellent (85-100)	8 (44.4)	5 (38.5)	3 (60.0)	0.61
Good (70-84)	10 (55.6)	8 (61.5)	2 (40.0)	0.61
Fair (50-69)	0 (0.0)	0 (0.0)	0 (0.0)	1.00
Poor (0-49)	0 (0.0)	0 (0.0)	0 (0.0)	1.00

DIP = distal interphalangeal, MCP = metacarpal phalangeal, PIP = proximal interphalangeal, TAM = total active motion

**Table 7:** Functional Outcomes of Digits With Zone II Injury Who Underwent Flexor Tendon Injuries Stratified by Mechanism of Injury

Variables	Saw Injury (N = 5)	Sharp Injury^a^ (N = 20)	*P* Value
Follow-up at 3 mo			
MCP (deg.)	85.2 ± 7.8	88.0 ± 1.2	0.12
PIP (deg.)	54.4 ± 7.2	64.9 ± 19.7	0.26
DIP (deg.)	31.8 ± 20.8	16.4 ± 10.9	0.03
TAM (%)	55.0 ± 12.1	46.9 ± 13.9	0.25
Dash score (no.)	15.8 ± 7.4	17.6 ± 9.2	0.69
Strickland criteria			
Excellent (85-100)	1 (20.0)	1 (5.0)	0.37
Good (70-84)	2 (40.0)	6 (30.0)	1.00
Fair (50-69)	2 (40.0)	11 (55.0)	0.64
Poor (0-49)	0 (0.0)	2 (10.0)	1.00
Follow-up at 6 mo			
MCP (deg.)	87.9 ± 2.6	89.3 ± 2.7	0.31
PIP (deg.)	78.8 ± 24.2	71.6 ± 19.6	0.49
DIP (deg.)	35.0 ± 17.8	39.8 ± 12.4	0.48
TAM (%)	74.9 ± 13.6	74.7 ± 10.2	0.97
Dash score (no.)	5.8 ± 3.5	11.0 ± 5.9	0.07
Strickland criteria			
Excellent (85-100)	3 (60.0)	8 (40.0)	0.62
Good (70-84)	2 (40.0)	12 (60.0)	0.62
Fair (50-69)	0 (0.0)	0 (0.0)	1.00
Poor (0-49)	0 (0.0)	0 (0.0)	1.00

DIP = distal interphalangeal, MCP = metacarpal phalangeal, PIP = proximal interphalangeal, TAM = total active motion

a Includes both knife/blade and glass-related lacerations.

## Discussion

This study prospectively analyses the progress of FDS repair and FDS excision for a 3-month and 6-month follow-up period after the repair of zone II flexor tendon injuries to compare functionality and disability in a Hispanic population. Currently, there is no consensus to help determine which primary surgical technique would have the best outcomes for Hispanics with zone II flexor tendon injuries. Our evaluation of the FDS-repair group at the 3-month follow-up revealed better functional outcomes and lower disability scores in comparison to the FDS-excision group. However, the 6-month evaluation between both groups did not reveal a notable difference. Therefore, we were not able to validate our hypothesis.

The tendon accommodations of both FDP and FDS tendons in a tight fibro-osseous tunnel remains one of the most important risk factors to predict poor surgical outcomes in zone II flexor tendon injuries.^3^ To minimize the bulk of the repaired tendon and facilitate tendon gliding, some surgeons advocate the approach of FDS tendon excision with repair of FDP alone, whereas others prefer the repair of both FDS and FDP tendons.^3,6^ Several studies have questioned the complete excision of FDS with FDP repair because of the devascularization effect of FDS tendon excision.^20,21^ Lister et al^30^ demonstrated that 85.7% of the digits who underwent repair of FDP and FDS tendons had excellent or good results compared with those who underwent FDS excision. The evaluation used at that moment was strictly based on the TAM score system, and recent evaluation systems such as the original Strickland criteria and DASH score were not performed. Further on, Nielsen and Jensen^7^ validated the importance of FDS repair in primary surgical repair of zone II injuries, where 74% of digits who underwent FDS repair had excellent or good results compared with only 47% on digits with FDS excision.

On the other hand, Zhao et al^5^ demonstrated through a biomechanical study that the excision of FDS tendon could decrease tendon gliding resistance under the A1 pulley in zone II flexor tendon repair. However, because of the circumstances of an in vitro study, their results were not able to measure postoperative functional outcomes and patient disabilities.^5^ In the same way, Tang^12^ proved that FDS excision could reduce the number of tendon sutures and eliminate possible adhesions between the repair of both FDP and FDS tendons. Although his results manifested TAM score improvement in Chinese patients with FDS excision, no notable difference was noted between both groups.^12^ Our results validate the study by Tang, conveying no difference regarding functional outcomes at the final follow-up visit in a Hispanic population.

The overall results of the TAM score and the original Strickland criteria in this study partially validates the assertion of past studies favoring the repair of both FDP and FDS tendon in the primary repair of zone II flexor tendon injuries.^1,21^ In our study, the comparison of TAM and Strickland criteria had markedly better outcomes toward the FDS tendon repair at the 3-month evaluation. However, the differences were not statistically significant when both groups were compared at the 6-months evaluation. The functional state of hand injuries, which are related to the well-being and satisfaction of every patient, was measured through the DASH questionnaire.^22^ The results provided in the questionnaire did not demonstrate a notable difference between both groups at the 6-month evaluation, although a notable difference was observed at the 3-month evaluation. Despite that, both groups showed very low disability scores. The authors of this study agree with previous studies that the DASH questionnaire focuses on multiple aspects of the upper extremity with questions that may be unrelated to the hand injury.^4,23^ Nevertheless, the results provided highlights the importance of satisfaction among Hispanics after treating upper extremity injuries.

The type of surgical procedure along with patient postoperative management are key factors that determine physical rehabilitation of hand injuries.^24^ The postoperative care of zone II flexor tendon injuries is one of the most challenging components of proper rehabilitation. Multiple studies have shown that zone II flexor tendon injuries should receive either earlier passive or earlier active passive ROM exercises as soon as they are repaired to prevent further complications such as stiffness, contractures and tenolysis.^15,21,25,26^ In 2005, Strickland^27^ showed no differences of tendon rupture rate between earlier passive and earlier active ROM exercises in patients with repaired flexor tendons. In our study, early passive ROM rehabilitation protocol was chosen due to primary investigator preference and past study experiences.^14,15,28^

The inclusion of a uniform protocol of early passive rehabilitation delivered by a single occupational therapist and the assurance of full access to physical therapy were fundamental in allowing the improvement of functional outcomes in both groups. This approach may be even more essential in Hispanic patients given that while time after injury increases, so do the differences in outcomes between different ethnic patient groups. These disparities may be a result of differences in the initiation and intensity of physical therapy in the postoperative period.^29^ Walsh et al^11^ determined that after either surgical or nonsurgical treatment of a distal radius fracture, Hispanic patients participated in less physical therapy sessions by 12 months than non-Hispanics, even after reporting poorer function and greater pain at most follow-ups. To maximize satisfactory outcomes, it is crucial to take into consideration unquantifiable factors such as cultural overtones, lifestyles, social contexts, acculturation, and incongruent value systems or cross-cultural miscommunication that may exist between patient and provider.^30,31^

Our study showed that Hispanic patients who underwent FDS excision had a tendon rupture rate of 8%, which is within the range of the typical rupture rate (between 4% and 10%) of four stranded cruciate flexor tendon repairs.^25^ FDP tendon rerupture was seen at the 3-month evaluation in two Hispanic patients. As described in other studies, the premature use of hand in active or resistive flexion could have explained the tendon rupture in these patients becoming nonadherent to therapy.^12,25^ This situation may also be secondary to an increased work of flexion among the single FDP tendon repaired. The comparison of complications between both groups did not reveal a notable difference despite that none of the 11 patients who underwent repair of both FDP and FDS tendon repair (FDS repair) had any postoperative complication. Despite revision surgery, the two patients were able to complete the protocol treatment without major complications.

The lack of statistical power was one of the main limitations of this study. The prospective observational nature of this study led to uneven sample sizes for the FDS-repair and FDS-excision groups. A factor that may have contributed to the lack of power may have been our strict inclusion criteria. Although flexor tendon injuries have been studied in-depth, they only represent less than 1% of acute hand injuries.^3,32^ Despite the low power of this study, our results showed notable difference with postoperative flexor functionality and disability assessment between both groups at their 3-month evaluation. To our knowledge, no data exist that shows the postoperative functionality and disability assessment of Hispanics who undergo zone II flexor tendon injuries.

## Conclusions

A trend toward higher functional outcomes and patient satisfaction, achieved with a standardized assessment, was demonstrated in both studied groups at the 6-month follow-up. Nevertheless, this study reported better functional outcomes with lower disability scores on digits of patients who underwent FDS repair compared with those who had excision of FDS tendon at the 3-month follow-up. These findings suggest that FDS tendon excision could have worse short-term satisfaction and functional outcomes after zone II repair compared with FDS tendon repair in a Hispanic population. Future studies are needed for the development of standardized guidelines to improve management of zone II flexor tendon injury.
